# Nutrition and mobility predict all-cause mortality in patients 12 months after transcatheter aortic valve implantation

**DOI:** 10.1007/s00392-017-1183-1

**Published:** 2017-11-21

**Authors:** Sarah Eichler, Annett Salzwedel, Axel Harnath, Christian Butter, Karl Wegscheider, Mihai Chiorean, Heinz Völler, Rona Reibis

**Affiliations:** 1 Center of Rehabilitation Research, University of Potsdam, Am Neuen Palais 10, House 12, 14469 Potsdam, Germany; 2 Sana Heart-Center Cottbus, Cottbus, Germany; 3 Heart Center Brandenburg in Bernau/Berlin and Brandenburg Medical School, Bernau, Germany; 4 Department of Medical Biometry and Epidemiology, University Medical Center, Hamburg-Eppendorf, Hamburg, Germany; 5 Klinik am See, Rehabilitation Center for Internal Medicine, Rüdersdorf, Germany; 6 Cardiological Outpatient Clinic Am Park Sanssouci, Potsdam, Germany

**Keywords:** TAVI, Frailty, Mortality, Malnutrition, Mobility

## Abstract

**Background:**

The aim of the study was to determine pre-interventional predictors for all-cause mortality in patients after transcatheter aortic valve implantation (TAVI) with a 12-month follow-up.

**Methods:**

From 10/2013 to 07/2015, 344 patients (80.9 ± 5.0 years, 44.5% male) with an elective TAVI were consecutively enrolled prospectively in a multicentre cohort study. Prior to the intervention, sociodemographic parameters, echocardiographic data and comorbidities were documented. All patients performed a 6-min walk test, Short Form 12 and a Frailty Index (score consisting of activities of daily living, cognition, nutrition and mobility). Peri-interventional complications were documented. Vital status was assessed over telephone 12 months after TAVI. Predictors for all-cause mortality were identified using a multivariate regression model.

**Results:**

At discharge, 333 patients were alive (in-hospital mortality 3.2%; *n* = 11). During a follow-up of 381.0 ± 41.9 days, 46 patients (13.8%) died. The non-survivors were older (82.3 ± 5.0 vs. 80.6 ± 5.1 years; *p* = 0.035), had a higher number of comorbidities (2.6 ± 1.3 vs. 2.1 ± 1.3; *p* = 0.026) and a lower left ventricular ejection fraction (51.0 ± 13.6 vs. 54.6 ± 10.6%; *p* = 0.048). Additionally, more suffered from diabetes mellitus (60.9 vs. 44.6%; *p* = 0.040). While the global Frailty Index had no predictive power, its individual components, particularly nutrition (OR 0.83 per 1 pt., CI 0.72–0.95; *p* = 0.006) and mobility (OR 5.12, CI 1.64–16.01; *p* = 0.005) had a prognostic impact. Likewise, diabetes mellitus (OR 2.18, CI 1.10–4.32; *p* = 0.026) and EuroSCORE (OR 1.21 per 5%, CI 1.07–1.36; *p* = 0.002) were associated with a higher risk of all-cause mortality.

**Conclusions:**

Besides EuroSCORE and diabetes mellitus, nutrition status and mobility of patients scheduled for TAVI offer prognostic information for 1-year all-cause mortality and should be advocated in the creation of contemporary TAVI risk scores.

## Introduction

The prevalence of aortic stenosis (AS) as the most frequent valve disease is still rising due to the demographic change and an aging population [1]. For patients with severe AS and a prohibitive surgical risk, transcatheter aortic valve implantation (TAVI) has been developed as an alternative to valve replacement, has reached widespread acceptance and is now used as a golden standard. Several clinical trials and registries have demonstrated the advantages and the procedural success of mid- to long-term outcomes [2, 3]. As the TAVI techniques become more reliable, procedural and in-hospital mortality rates could be reduced. Therefore, the frequency of TAVI procedures is steadily increasing and has even overtaken the slightly decreased number of surgical procedures in Germany [4].

Nevertheless, due to the medium- to high-risk population, the 1-year mortality rate is still high at about 15–20% [5]. Several clinical prediction models including the surgical EuroSCORE, the Society of Thoracic Surgeons (STS) score as well as the more TAVI-specific FRANCE-2 [6], German Aortic Valve [7] and the OBSERVANT score [8] were established to estimate the peri-interventional risk. However, besides conventional risk factors including age, reduced systolic left ventricular function and renal failure, which are captured in conventional risk scores, little is known about reliable functional prognostic factors for procedural and 1-year mortality. While reduced baseline performance status is a well-established predictor of operative risk [5], an overall frailty assessment should be particularly relevant for patients with severe aortic stenosis referred to TAVI.

Regarding the future needs of clinical trials, the Valve Academic Research Consortium defined frailty as a multicomponent parameter including the criteria of slowness, weakness, exhaustion, wasting and malnutrition, poor endurance and inactivity as well as a loss of independence [9]. Until now, frailty has not been considered as an independent cardiovascular risk factor and has not been incorporated into traditional risk scores and recent clinical prediction models. Furthermore, several different approaches for measuring frailty have been described. Two indices seem to prevail in clinical studies [10, 11], but it remains unknown which of the many frailty indices best predicts outcomes such as mortality, especially in TAVI patients.

The aim of the present study was to evaluate pre-interventional predictors, escpecially frailty-related parameters not captured by traditional risk scores for 1-year all-cause mortality in patients after TAVI.

## Methods

### Study setting and participants

**Fig. 1:**
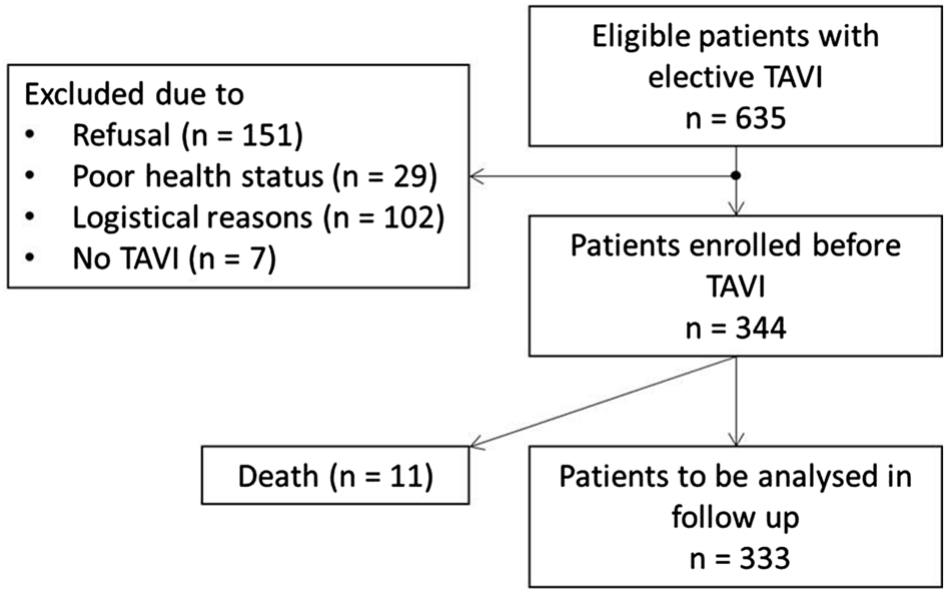
CONSORT Flow chart of inclusion process. Logistical reasons for exclusion: due to short-term intervention date shifts, the research fellows of the University of Potsdam were unable to perform the functional and frailty assessments in the heart centres

In this prospective multicentre cohort study, 635 patients referred for elective TAVI were screened in two German heart centres between October 2013 and July 2015. After the exclusion of 291 patients, 344 patients scheduled for TAVI could be enrolled prior to the procedure; 333 (96.8%) patients were alive at discharge (Fig. 1).

The study protocol was approved by the ethics committee of the University of Potsdam (No. 35/2013). All patients gave their written consent to participate in the investigation. Data protection rules were closely observed and patient data were processed anonymously.

### Baseline measures and clinical data

Sociodemographic data (e.g. age and gender), comorbidities [e.g. chronic obstructive pulmonary disease (COPD) and chronic kidney disease (CKD)] and echocardiographic data [e.g. left ventricular ejection fraction (LVEF) and transaortic gradients] as well as the peri- and postprocedural complications according to the VARC-2 criteria [9] were taken from the patients’ records.

For the quantification of the performance status and the health-related quality of life a standardized 6-min walk test (6MWT) according to current guidelines of the American Thoracic Society [12] based on a distance measuring device was performed. Before starting the test, the patient was familiarized with the procedure to be followed. In addition, the questionnaire Short Form 12 (SF-12) with its physical and mental component summaries (PCS and MCS) [13] was assessed. Anxiety and depression were objected using the Hospital Anxiety and Depression Scale (HADS) [14], and frailty according to the index of Stortecky et al. [15]. This Frailty Index includes the Mini-Mental State Examination (MMSE), the short form of the Mini Nutritional Assessment (MNA), activities of daily living (ADL), instrumental activities of daily living (IADL), timed-up-and-go test (TUG) and a subjective mobility disability (defined as a decreased frequency of walking 200 m and/or of climbing stairs). The index was summarised with the following allocations: 2 points were assigned if MMSE was < 21 points, and 1 point was assigned for each of the following: MMSE ≥ 21 and < 27 points, MNA < 12 points, ADL ≥ 1 limited activity, IADL ≥ 1 limited activity, TUG ≥ 20 s, and a positive subjective mobility disability. Hence, the Frailty Index ranged from 0 to 7 points and can be categorised at ≥ 3 points (frail) vs. < 3 points (non-frail). All functional and frailty tests were assessed in the two heart centres by trained research fellows of the University of Potsdam.

### Follow-up

The primary endpoint was all-cause mortality at 12 months after TAVI. The patients’ vital status was assessed over the telephone. If no reply was received, the family doctor and the treating hospitals were contacted by telephone. If still no information was received, vital status was established via the residents’ registry office. There were no patients lost to follow-up (100% follow-up rate).

### Statistics

Continuous variables are expressed as means ± standard deviation (SD), and categorical variables as absolute values and percentages. Comparisons between groups were performed using the *t* test and the Chi-square test, respectively. Predictors for all-cause mortality were identified using a multivariate logistic regression model. We started with a full model containing all available covariates and performed a backwards selection to keep only significant effects in the model. In the first step, following variables were included: sex, age, body mass index, physical activity, living situation, care dependency, graduation, New York Heart Association (NYHA) class III/IV, diabetes mellitus, EuroSCORE, coronary artery disease, COPD, pacemaker, peripheral artery disease, aortic aneurysm, stroke or transient ischemic attack, CKD, hepatic dysfunction, carcinoma, osteoarthritis, LVEF < 40%, mitral regurgitation, resting heart rate, atrial fibrillation, QRS width, block, hemoglobin, glomerular filtration rate, SF-12, anxiety, depression, MNA, ADL, IADL, MMSE, mobility disability, TUG, Frailty Index, 6MWT as well as a number of comorbidities. Effects with a *p* value of less than 0.05 (two-sided) were considered significant. Calculations were carried out using SPSS 23.0 (IBM, Chicago, IL, USA).

## Results

### Peri-/postprocedural outcomes

The mean age of the patients was 80.9 ± 5.0 years, 55.5% were women. Pacemakers were implanted in 43 patients (12.5%); major vascular complications were detected in 28 cases (8.1%), while 11 patients died (all-cause in-hospital mortality 3.2%). A conversion to open surgery was necessary in eight patients (Table 1).

**Table 1:** Peri-/postprocedural and in-hospital outcomes (*n* = 344)

Variable	*N* (%)	
All-cause mortality	11 (3.2)	
Cardiovascular mortality	6 (1.7)	
Non-cardiovascular mortality	5 (1.5)	
Stroke/TIA	7 (2.0)	
Major bleeding	15 (4.4)	
Acute kidney injury	9 (2.6)	
Major vascular complications	28 (8.1)	
Endovascular stenting	17 (4.9)	
Surgical repair	11 (3.2)	
Pacemaker implantation	43 (12.5)	
Conversion to open surgery	8 (2.3)	
Infection/pneumonia	7 (2.0)	

*TIA* transient ischemic attack

### Baseline data (total cohort)

Most of the patients had NYHA class III or IV (96.7%). Almost half of the patients suffered from diabetes mellitus (46.8%). The mean left ventricular ejection fraction was 54.1 ± 11.1%. Maximum and mean transvalvular aortic gradients were 71.3 ± 25.4 and 44.8 ± 16.7 mmHg, respectively. Patients had a mean logistic EuroSCORE of 16.9 ± 11.9% and 2.2 ± 1.3 comorbidities. The in-hospital stay was 10.9 ± 4.6 days. (Table 2). TAVI was performed under a short period of general anaesthesia in 128 (38.4%) patients and local anaesthesia in 205 (61.6%) patients. The main access route was through the femoral artery in 319 (95.8%) patients and via a left-sided small anterolateral minithoracotomy in 14 (4.2%) patients. A Medtronic CoreValve^®^ classic and CoreValve^®^ Evolut R Prosthesis (Medtronic Inc., Minnesota, USA) was implanted in 181 (54.4%) and 24 (7.2%) patients, an Edwards SAPIEN 3^™^ or SAPIEN XT^™^ transcatheter heart valve (Edwards Lifesciences LLC, Irvine, CA, USA) in 80 (24.0%) and 10 (3.0%) patients, respectively. Other prostheses were used in 38 (11.4%) patients.

**Table 2:** Baseline characteristics of post-procedural population (total cohort, survivors vs. non-survivors)

	Total (*n* = 333)	Survivors (*n* = 287)	Non-survivors (*n* = 46)	*p* value
Patients’ characteristics				
Age, years	80.8 ± 5.1	80.6 ± 5.1	82.3 ± 5.0	0.035
Sex, male	147 (44.1)	125 (43.6)	22 (47.8)	0.350
NYHA III/IV	322 (96.7)	277 (96.5)	45 (97.8)	0.644
Diabetes mellitus	156 (46.8)	128 (44.6)	28 (60.9)	0.040
Log. EuroSCORE, %	16.9 ± 11.9	15.7 ± 10.2	23.9 ± 17.8	< 0.001
Comorbidities, no.	2.2 ± 1.3	2.1 ± 1.3	2.6 ± 1.3	0.026
COPD	62 (18.6)	50 (17.4)	12 (26.1)	0.161
PAD	72 (21.6)	57 (19.9)	15 (32.6)	0.051
CKD	159 (47.7)	129 (44.9)	30 (65.2)	0.011
Length of hospital stay, days	10.9 ± 4.6	10.6 ± 4.4	13.0 ± 5.5	0.001
2D Echocardiography				
LVEF, %	54.1 ± 11.1	54.6 ± 10.6	51.0 ± 13.6	0.048
Left atrium, mm	45.3 ± 6.3	45.0 ± 6.3	47.0 ± 6.3	0.068
LVEDD	48.1 ± 8.2	47.9 ± 7.9	49.3 ± 9.6	0.278
LVPW	13.3 ± 3.0	13.3 ± 3.0	13.0 ± 2.6	0.453
IVS	13.4 ± 2.7	13.4 ± 2.7	13.3 ± 2.6	0.783
Transaortic ∆ *P*_mean_ (mmHg)	44.8 ± 16.7	45.6 ± 17.0	39.6 ± 14.3	0.025
Transaortic ∆ *P*_max_ (mmHg)	71.3 ± 25.4	72.4 ± 25.8	63.9 ± 21.6	0.039

Categorical variables are presented in n (%), metric variables in mean ± SD

*COPD* chronic obstructive pulmonary disease, *PAD* peripheral artery disease, *CKD* chronic kidney disease, *LVEF* left ventricular ejection fraction, *LVEDD* left ventricular end-diastolic diameter, *LVPW* left ventricular posterior wall, *IVS* interventricular septum

Prior to TAVI, the patients achieved a 6-min walk distance (6MWD) of 230.1 ± 119.1 m. Patients needed 14.2 ± 7.0 s. in the TUG. The mean Frailty Index was 2.5 ± 1.7 points (non-frail) (Table 3).

**Table 3:** Baseline assessments of post-procedural population (total cohort, survivors vs. non-survivors)

Assessments	Total (*n* = 333)	Survivors (*n* = 287)	Non-survivors (*n* = 46)	*p* value
6MWD, m	230.1 ± 119.1	237.1 ± 122.0	179.2 ± 81.2	0.020
Quality of life				
SF-12 PCS, points	33.2 ± 9.9	33.6 ± 9.8	30.5 ± 9.7	0.050
SF-12 MCS, points	50.8 ± 10.4	50.9 ± 10.4	49.8 ± 10.6	0.475
Emotional status				
HADS anxiety, points	5.4 ± 3.7	5.4 ± 3.7	5.7 ± 3.7	0.552
HADS Depression, points	5.5 ± 3.7	5.3 ± 3.5	6.9 ± 4.4	0.007
Frailty Index, points	2.5 ± 1.7	2.4 ± 1.6	3.3 ± 1.7	< 0.001
Frailty Index ≥ 3 points	152 (45.8)	122 (42.7)	30 (65.2)	0.004
MMSE, points	26.8 ± 3.0	26.9 ± 3.0	26.1 ± 3.1	0.074
MNA, points	11.7 ± 2.3	11.9 ± 2.2	10.7 ± 2.5	0.001
ADL, points	93.2 ± 12.7	93.5 ± 12.5	91.6 ± 13.5	0.355
IADL, points	6.9 ± 1.7	7.0 ± 1.7	6.5 ± 1.5	0.105
TUG, s	14.2 ± 7.0	13.8 ± 7.2	16.4 ± 5.1	0.026
Mobility disability	255 (76.8)	217 (75.9)	38 (82.6)	0.315

Categorical variables are presented in n (%), metric variables in mean ± SD

*6MWD* 6-min walk distance, *SF-12* Short Form 12, *PCS* physical component summary, *MCS* mental component summary, *HADS* Hospital Anxiety and Depression Scale, *MMSE* Mini-Mental State Exam, *MNA* Mini Nutritional Assessment, *ADL* activities of daily living, *IADL* instrumental activities of daily living, *TUG* timed-up-and-go test

### Survivors vs. non-survivors

During a follow-up of 381.0 ± 41.9 days, 46 patients (13.8%) died. In the univariate analysis, the non-survivors were older (82.3 ± 5.0 vs. 80.6 ± 5.1 years; *p* = 0.035), had a higher number of comorbidities (2.6 ± 1.3 vs. 2.1 ± 1.3; *p* = 0.026) and a lower left ventricular ejection fraction (51.0 ± 13.6 vs. 54.6 ± 10.6%; *p* = 0.048). The maximum and mean transvalvular aortic gradients were lower in the non-survivors (63.9 ± 21.6 vs. 72.4 ± 25.8; *p* = 0.039 and 39.6 ± 14.3 vs. 45.6 ± 17.0; *p* = 0.025, respectively).

Additionally, more patients suffered from diabetes mellitus (60.9 vs. 44.6%; *p* = 0.040) and chronic kidney disease (65.2 vs. 44.9%, *p* = 0.011) and had a longer stay in the hospital (13.0 ± 5.5 vs. 10.6 ± 4.4 days; *p* = 0.001).

Likewise, the patient groups differed in several assessment parameters. The non-survivors had worse results in the 6MWD (179.2 ± 81.2 vs. 237.1 ± 122.0 m, *p* = 0.020), in the Frailty Index [3.3 ± 1.7 (frail) vs. 2.4 ± 1.6 points (non-frail), *p* < 0.001] and in individual components of the Frailty Index: TUG (16.4 ± 5.1 vs. 13.8 ± 7.2 s; *p* = 0.026) and MNA (10.7 ± 2.5 vs. 11.9 ± 2.2 points, *p* = 0.001). Furthermore, PCS was significantly lower in the non-survivors (30.5 ± 9.7 vs. 33.6 ± 9.8; *p* = 0.050), while depression was higher (6.9 ± 4.4 vs. 5.3 ± 3.5; *p* = 0.007) (Table 2).

### Predictors for all-cause mortality

**Fig. 2:**
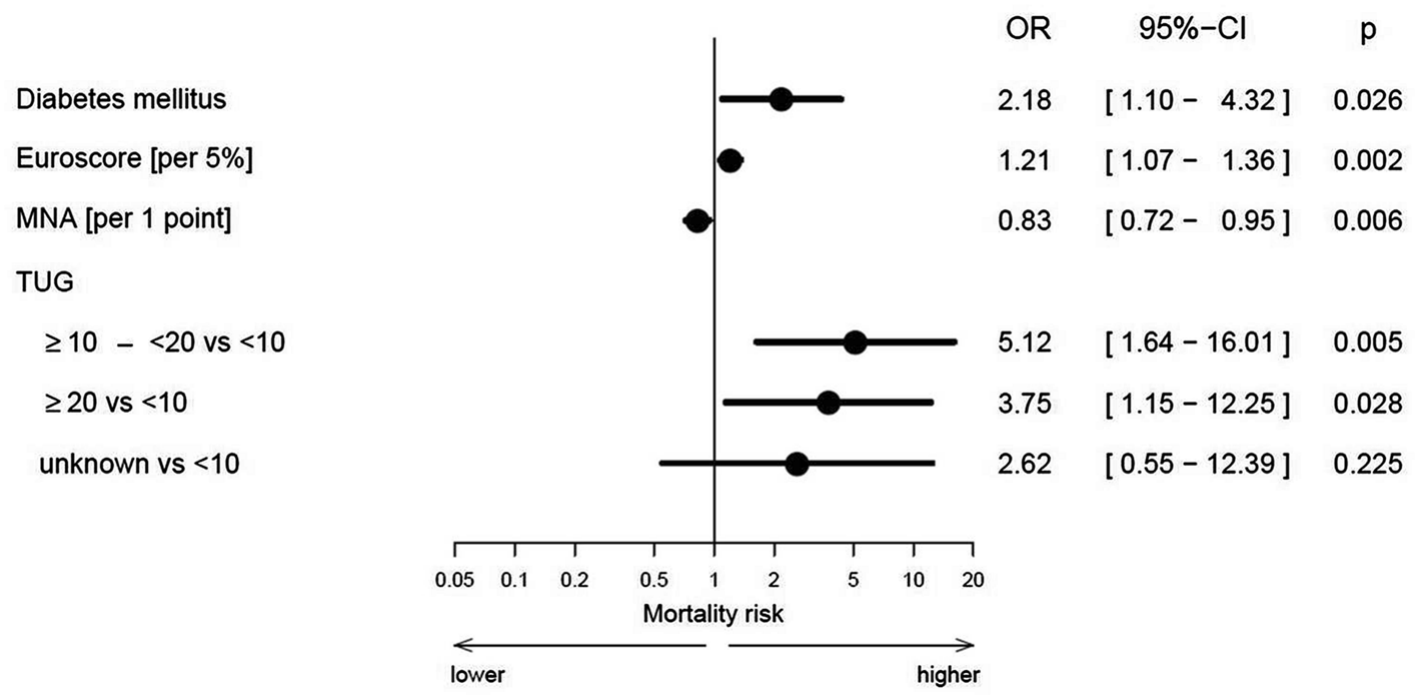
Predictors for 1-year all-cause mortality (*n* = 333). *OR* odds ratio, *CI* confidence interval, *MNA* Mini Nutritional Assessment, *TUG* timed-up-and-go test

In the multivariate analysis, the Frailty Index was not predictive for all-cause mortality, but individual components such as nutrition (MNA: OR 0.83 per 1 point, CI 0.72–0.95; *p* = 0.006) and mobility (TUG ≥ 10–< 20 vs. < 10 s: OR 5.12, CI 1.64–16.01; *p* = 0.005) revealed prognostic impact (Fig. 2).

Furthermore, clinical parameters like diabetes mellitus (OR 2.18, CI 1.10–4.32; *p* = 0.026) and EuroSCORE (OR 1.21 per 5%, CI 1.07–1.36; *p* = 0.002) were associated with a higher risk of all-cause mortality (Fig. 2).

## Discussion

Our analysis supports the finding that pre-interventional nutrition state and global mobility reveal predictive power for 1-year all-cause mortality in TAVI patients. Additionally, clinical parameters including diabetes mellitus and the logistic EuroSCORE, initially used to estimate the operative risk in surgical patients, were objected as prognostic parameters. While these last parameters go along with conventional prognostic parameters including lower left ventricular ejection fraction, chronic kidney disease and advanced age, and are already extensively studied, the frailty parameters are still underused in the estimation of peri-procedural risk for patients scheduled for TAVI procedures [16, 17].

Recent studies have confirmed the strong association between frailty state and the 12-month mortality [18, 19]. Several clinical prediction models for TAVI have been suggested [6–8, 20], so that the “eyeball test” does not need to be used. Nevertheless, despite including multiple parameters, the predictive power is still rather inadequate [21]. Thus, there is an urgent need for the characterization of potentially threatened patients, including readmission [22] and all-cause mortality [23].

Our cohort represents a large scale group of patients in two German Heart Centers, who were examined prior to TAVI through performance and health-related quality of life tests. We assessed a 6-min walk test, a questionnaire Short Form 12 and Anxiety and Depression Scale. Also documented, the Frailty Index of Stortecky [15] represents an extensive objectification of general functioning, including psycho-cognitive, nutritive and physical components. Alternatively, the semi-quantitative Clinical Frailty Scale (CFS) includes other indications of frailty, like serum albumin, body mass index, gait speed and mean hand grip, and is an independent predictive factor of increased cumulative mortality risk as well [19].

Frailty is a geriatric syndrome, which is characterized by a vulnerable health status associated with declining function of multiple physiological systems and loss of physiological reserves with consecutive impairment of many domains (physical, social, nutritional, neuropsychological) [24, 25]. The presence of frailty has been associated with poor medical outcomes in different cardiac patients such as patients with coronary artery disease [26] and chronic heart failure [27] as well as in patients undergoing cardiac surgery or TAVI. In 2012, Stortecky et al. suggested a multidimensional geriatric assessment for TAVI candidates with a solid association with all-cause mortality and major cardiovascular events at 30 days and at 1-year follow-up [15].

However, as this multifactorial, frailty is difficult to capture and it is time-consuming to assess all components; it might be difficult to implement this test in clinical practice. As an alternative, the consideration of single components seems to be useful. In our analysis, we could confirm that nutrition and mobility as individual components of frailty, but not the global index, have a predictive value for all-cause mortality in patients after TAVI. The Mini Nutritional Assessment has been used as a screening tool for different entities, particularly in hospitalised patients at advanced age. We could demonstrate that, for each additional point in the MNA, patients had a reduced mortality risk of 17% within 12 months. These data go along with earlier findings showing a strong association between malnutrition and worse outcome [28].

Whereas overweight can lead to a better prognosis in patients with cardiovascular diseases and is the called “obesity paradox” [29], malnutrition and the malabsorption of nutrition are serious health problems in the elderly and can have a negative influence on function and quality of life [30]. Recent research has already shown the prognostic relevance of malnutrition in other populations, such as acute heart failure due to left ventricular systolic dysfunction [17]. When recognized early on, malnutrition can be reversible [31] by performing a suitable nutritive intervention. Therefore, patients should be screened before TAVI and, when appropriate, cared for to improve the outcome.

Furthermore, mobility has been proven to be an important prognostic factor in elderly patients with different cardiovascular diseases or in patients undergoing cardiac procedures [32]. To assess mobility, the study used the gait speed test, which is easy and quick to implement in clinical practice when investigating TAVI patients before their interventions. The timed-up-and-go test used in this study is quick to implement as well, can be performed by assistant personnel and requires no additional instruments. Our data suggest that for those patients who needed ≥ 10–< 20 s for the TUG, the all-cause mortality risk was 5.12 times higher in comparison to those, whose mobility was better and consecutively needed fewer than 10 s. In the multivariate analysis, this simple test had the most certain predictive ability, while all conventional clinical data including LVEF, valve-related echocardiographic parameters, renal function and age showed no prognostic impact, though it might have been expected.

When a patient is limited in his mobility, different training strategies focusing on strength, coordination and balance should be performed after TAVI, also considering fall prevention in the elderly. It is also conceivable that this training can be performed even before the intervention as there already exist different approaches involving a training program in cardiac surgery patients even before a surgery or an intervention, which is called prehabilitation, to improve the outcome [33]. Until now, the evidence for specific pre-operative nutrition programs focusing on prognostic parameters is still weak. However, depending on the stability of aortic stenosis, it appears desirable to optimise the global mobility and nutrition situation in advance of the operation in order to improve the immediate interventional success and the further prognosis of the patients.

### Limitations

The present study has certain limitations. First, participation in the cohort study was voluntary and thus not without a selection bias, particularly in patients with higher risk profiles. Due to short-term intervention date shifts, the research fellows of the University of Potsdam were unable to perform the functional and frailty assessments in the heart centres in 102 cases. Since there was no link between intervention date shifts and 1-year outcome the high drop-out rate did not bias the study results; it only resulted in a loss of power. Further, we do not take into account information about the clinical course of the TAVI procedure, including procedure time, peri-interventional pharmacotherapy and intensive care unit stay, all of which can have an influence on the dynamic of the functional improvement. Additionally, we do not have post-procedural echocardiographic data regarding the quality of the valve implantation, which can affect the clinical outcome as well. Although the Stortecky’s Frailty Index captures components such as nutrition, it would be of interest to differentiate between lean and fat body mass as an index of sarcopenia. This would require a further approach to characterising TAVI patients and may be advocated for detailed research.

## Conclusion

The results provide information about pre-interventional frailty parameters being predictive for 1-year all-cause mortality in patients after TAVI. Particularly, nutrition status and mobility should be advocated in the creation of contemporary TAVI risk scores.

## References

[1] Zahn R, Werner N, Gerckens U, Linke A, Sievert H, Kahlert P, Hambrecht R, Sack S, Abdel-Wahab M, Hoffmann E, Zeymer U, Schneider S (2017). Five-year follow-up after transcatheter aortic valve implantation for symptomatic aortic stenosis. Heart.

[2] Siontis GC, Praz F, Pilgrim T, Mavridis D, Verma S, Salanti G, Sondergaard L, Juni P, Windecker S (2016). Transcatheter aortic valve implantation vs. surgical aortic valve replacement for treatment of severe aortic stenosis: a meta-analysis of randomized trials. Eur Heart J.

[3] Smith CR, Leon MB, Mack MJ, Miller DC, Moses JW, Svensson LG, Tuzcu EM, Webb JG, Fontana GP, Makkar RR, Williams M, Dewey T, Kapadia S, Babaliaros V, Thourani VH, Corso P, Pichard AD, Bavaria JE, Herrmann HC, Akin JJ, Anderson WN, Wang D, Pocock SJ, Investigators PT (2011). Transcatheter versus surgical aortic-valve replacement in high-risk patients. N Engl J Med.

[4] Gaede L, Blumenstein J, Kim W-K, Liebetrau C, Dörr O, Nef H, Hamm C, Elsässer A, Möllmann H (2017). Trends in aortic valve replacement in Germany in 2015: transcatheter versus isolated surgical aortic valve repair. Clin Res Cardiol.

[5] Zahn R, Gerckens U, Linke A, Sievert H, Kahlert P, Hambrecht R, Sack S, Abdel-Wahab M, Hoffmann E, Schiele R, Schneider S, Senges J, German Transcatheter Aortic Valve Interventions-Registry I (2013). Predictors of one-year mortality after transcatheter aortic valve implantation for severe symptomatic aortic stenosis. Am J Cardiol.

[6] Iung B, Laouenan C, Himbert D, Eltchaninoff H, Chevreul K, Donzeau-Gouge P, Fajadet J, Leprince P, Leguerrier A, Lievre M, Prat A, Teiger E, Laskar M, Vahanian A, Gilard M, for the FI (2014). Predictive factors of early mortality after transcatheter aortic valve implantation: individual risk assessment using a simple score. Heart.

[7] Kotting J, Schiller W, Beckmann A, Schafer E, Dobler K, Hamm C, Veit C, Welz A (2013). German Aortic Valve Score: a new scoring system for prediction of mortality related to aortic valve procedures in adults. Eur J Cardio Thorac Surg.

[8] Capodanno D, Barbanti M, Tamburino C, D’Errigo P, Ranucci M, Santoro G, Santini F, Onorati F, Grossi C, Covello RD, Capranzano P, Rosato S, Seccareccia F, Group OR (2014). A simple risk tool (the OBSERVANT score) for prediction of 30-day mortality after transcatheter aortic valve replacement. Am J Cardiol.

[9] Kappetein AP, Head SJ, Genereux P, Piazza N, van Mieghem NM, Blackstone EH, Brott TG, Cohen DJ, Cutlip DE, van Es GA, Hahn RT, Kirtane AJ, Krucoff MW, Kodali S, Mack MJ, Mehran R, Rodes-Cabau J, Vranckx P, Webb JG, Windecker S, Serruys PW, Leon MB (2012). Updated standardized endpoint definitions for transcatheter aortic valve implantation: the Valve Academic Research Consortium-2 consensus document. Eur Heart J.

[10] Rockwood K, Mitnitski A (2007). Frailty in relation to the accumulation of deficits. J Gerontol Ser A Biol Sci Med Sci.

[11] Fried LP, Tangen CM, Walston J, Newman AB, Hirsch C, Gottdiener J, Seeman T, Tracy R, Kop WJ, Burke G, McBurnie MA, Cardiovascular Health Study Collaborative Research G (2001). Frailty in older adults: evidence for a phenotype. J Gerontol Ser A Biol Sci Med Sci.

[12] Laboratories ACoPSfCPF (2002). ATS statement: guidelines for the six-minute walk test. Am J Respir Crit Care Med.

[13] Ware J, Kosinski M, Keller SD (1996). A 12-item short-form health survey: construction of scales and preliminary tests of reliability and validity. Med Care.

[14] Hinz A, Brahler E (2011). Normative values for the hospital anxiety and depression scale (HADS) in the general German population. J Psychosom Res.

[15] Stortecky S, Schoenenberger AW, Moser A, Kalesan B, Juni P, Carrel T, Bischoff S, Schoenenberger CM, Stuck AE, Windecker S, Wenaweser P (2012). Evaluation of multidimensional geriatric assessment as a predictor of mortality and cardiovascular events after transcatheter aortic valve implantation. JACC Cardiovasc Interv.

[16] Wada H, Dohi T, Miyauchi K, Doi S, Naito R, Konishi H, Tsuboi S, Ogita M, Kasai T, Hassan A, Okazaki S, Isoda K, Suwa S, Daida H (2017). Prognostic impact of the geriatric nutritional risk index on long-term outcomes in patients who underwent percutaneous coronary intervention. Am J Cardiol.

[17] Sze S, Zhang J, Pellicori P, Morgan D, Hoye A, Clark AL (2017). Prognostic value of simple frailty and malnutrition screening tools in patients with acute heart failure due to left ventricular systolic dysfunction. Clin Res Cardiol.

[18] Kleczynski P, Dziewierz A, Bagienski M, Rzeszutko L, Sorysz D, Trebacz J, Sobczynski R, Tomala M, Stapor M, Dudek D (2017). Impact of frailty on mortality after transcatheter aortic valve implantation. Am Heart J.

[19] Shimura T, Yamamoto M, Kano S, Kagase A, Kodama A, Koyama Y, Tsuchikane E, Suzuki T, Otsuka T, Kohsaka S, Tada N, Yamanaka F, Naganuma T, Araki M, Shirai S, Watanabe Y, Hayashida K, Investigators O-T (2017). Impact of the clinical frailty scale on outcomes after transcatheter aortic valve replacement. Circulation.

[20] O’Brien SM, Shahian DM, Filardo G, Ferraris VA, Haan CK, Rich JB, Normand SL, DeLong ER, Shewan CM, Dokholyan RS, Peterson ED, Edwards FH, Anderson RP (2009). The Society of Thoracic Surgeons 2008 cardiac surgery risk models: part 2—isolated valve surgery. Ann Thorac Surg.

[21] Martin GP, Sperrin M, Ludman PF, de Belder MA, Gale CP, Toff WD, Moat NE, Trivedi U, Buchan I, Mamas MA (2017). Inadequacy of existing clinical prediction models for predicting mortality after transcatheter aortic valve implantation. Am Heart J.

[22] Franzone A, Pilgrim T, Arnold N, Heg D, Langhammer B, Piccolo R, Roost E, Praz F, Raber L, Valgimigli M, Wenaweser P, Juni P, Carrel T, Windecker S, Stortecky S (2017). Rates and predictors of hospital readmission after transcatheter aortic valve implantation. Eur Heart J.

[23] Chandrasekhar J, Dangas G, Yu J, Vemulapalli S, Suchindran S, Vora AN, Baber U, Mehran R, Registry SAT (2016). Sex-based differences in outcomes with transcatheter aortic valve therapy: TVT registry from 2011 to 2014. J Am Coll Cardiol.

[24] Afilalo J, Alexander KP, Mack MJ, Maurer MS, Green P, Allen LA, Popma JJ, Ferrucci L, Forman DE (2014). Frailty assessment in the cardiovascular care of older adults. J Am Coll Cardiol.

[25] Clegg A, Young J, Iliffe S, Rikkert MO, Rockwood K (2013). Frailty in elderly people. Lancet.

[26] Nunez J, Ruiz V, Bonanad C, Minana G, Garcia-Blas S, Valero E, Nunez E, Sanchis J (2017). Percutaneous coronary intervention and recurrent hospitalizations in elderly patients with non ST-segment acute coronary syndrome: The role of frailty. Int J Cardiol.

[27] Denfeld QE, Winters-Stone K, Mudd JO, Hiatt SO, Chien CV, Lee CS (2017). Frequency of and significance of physical frailty in patients with heart failure. Am J Cardiol.

[28] Liu GX, Chen Y, Yang YX, Yang K, Liang J, Wang S, Gan HT (2017). Pilot study of the mini nutritional assessment on predicting outcomes in older adults with type 2 diabetes. Geriatr Gerontol Int.

[29] Oga EA, Eseyin OR (2016). The obesity paradox and heart failure: a systematic review of a decade of evidence. J Obes.

[30] Marshall S, Bauer J, Isenring E (2014). The consequences of malnutrition following discharge from rehabilitation to the community: a systematic review of current evidence in older adults. J Hum Nutr Diet.

[31] Posner BM, Jette AM, Smith KW, Miller DR (1993). Nutrition and health risks in the elderly: the nutrition screening initiative. Am J Public Health.

[32] Afilalo J, Kim S, O’Brien S, Brennan JM, Edwards FH, Mack MJ, McClurken JB, Cleveland JC, Smith PK, Shahian DM, Alexander KP (2016). Gait speed and operative mortality in older adults following cardiac surgery. JAMA Cardiol.

[33] Hulzebos EH, Smit Y, Helders PP, van Meeteren NL (2012). Preoperative physical therapy for elective cardiac surgery patients. Cochrane Database Syst Rev.

